# Supplementary material for: Tailoring the coercive field in ferroelectric metal-free perovskites by hydrogen bonding
Source: Nat Commun. 2022 Feb 10;13:794. doi: 10.1038/s41467-022-28314-8 (PMC8831526; doi:10.1038/s41467-022-28314-8)
Supplement: Supplementary file 1 — Supplementary Information [file 41467_2022_28314_MOESM1_ESM.pdf]

Supplementary Information for

Tailoring the Coercive Field in Ferroelectric Metal-Free Perovskites by Hydrogen Bonding

Hwa Seob Choi[1,7], Shunning Li[2,7], In-Hyeok Park[3], Weng Heng Liew[4], Ziyu Zhu[5], Ki Chang Kwon[1,5], Lin Wang[5], In-Hwan Oh[6], Shisheng Zheng[2], Chenliang Su[1], Qing-Hua Xu[5], Kui Yao[4], Feng Pan[2]*, Kian Ping Loh,[1,5]*

[1]SZU-NUS Collaborative Innovation Center for Optoelectronic Science & Technology, International Collaborative Laboratory of 2D Materials for Optoelectronics Science and Technology of Ministry of Education, Institute of Microscale Optoelectronics, Shenzhen University, 518060 Shenzhen, P.R. China.

[2]School of Advanced Materials, Peking University Shenzhen Graduate School, 518055 Shenzhen, P.R. China.

[3]Graduate School of Analytical Science and Technology (GRAST), Chungnam National University, Daejeon 34134, Republic of Korea.

*[4]Institute of Materials Research and Engineering, A*STAR (Agency for Science, Technology and Research), 2 Fusionopolis Way, 138634 Singapore, Singapore.*

[5]Department of Chemistry, National University of Singapore, 3 Science Drive 3, 117543 Singapore, Singapore.

[6]Neutron Science Division, Korea Atomic Energy Research Institute, Daejeon 34057, Republic of Korea.

[7]*These authors contributed equally: Hwa Seob Choi, Shunning Li*

*Corresponding authors: panfeng@pkusz.edu.cn (F.P.); chmlohkp@nus.edu.sg (K.P.L.)

Method details

Chemicals and characterization

All chemicals were purchased from Sigma-Aldrich without further purification. ^{1}H and ^{13}C nuclear magnetic resonance (NMR) was taken by AVII 400 MHz NMR spectrometer of Bruker. Thermogravimetric analyses (TGA) were performed under a nitrogen atmosphere with a heating rate of 10 °C/min using a TA Instruments Trios V3.1 thermogravimetric analyzer. Differential scanning calorimetry (DSC) scans were performed under a nitrogen atmosphere with a heating rate of 10 °C/min using Mettler-Toledo DSC. The dielectric constant was measured by the CVU unit in Keithley-SCS4200 with the pelleted sample. Powder X-ray diffraction (PXRD) patterns were recorded on a Bruker D8 Focus Powder X-ray diffractometer using Cu Kα radiation (40 kV, 40 mA) at room temperature. Ferroelectric P-E curve was measured with Precision Multiferroic II Ferroelectric Test System of the Radiant Technologies with high voltage amplifier. Piezoresponse force microscopy (PFM) tests were performed on Bruker Dimension Icon Atomic Force Microscope with grown crystals spray-coated samples on ITO.

Single-crystal X-ray analysis

The single-crystal X-ray diffraction (SC-XRD) intensity data for MNP_3 were measured at room (T = 298 K) and high temperature (T = 353 K), using a four circle goniometer with Kappa geometry and Bruker AXS D8 Venture Single Crystal X-ray Diffractometer equipped with a Photon 100 CMOS active pixel sensor detector. Data collection, data reduction, and absorption correction were carried out using the software package of APEX3. All calculations for structure determination were carried out using the SHELXTL package 3. Data are available from the corresponding author on request. CCDC- 2085249 (MNP_3 at RT) and CCDC-2085250 (D-MNP_3 at RT) contain the supplementary crystallographic data for this paper. These data can be obtained free of charge from the Cambridge Crystallographic Data Centre via www.ccdc.cam.ac.uk/data_request/cif.

Second-harmonic generation (SHG)

For SHG measurement, a home-built optical set-up was assembled with a microscope (Nikon, Eclipse Ti) to measure the SHG of crystal samples. The laser excitation source with 140 fs pulses, generated by a Ti:sapphire oscillator, has a repetition rate at 80 MHz and a central wavelength at 800 nm. The 800 nm laser beam was cleaned by one 785 nm long-pass filter (Semrock, LP02-785RU-25) fixed before the microscope. The perovskite crystals were settled on a 3-D piezo stage (PI P-545). A 10 times objective lens (NA 0.3) focused the beam and collected the SHG signal. The SHG signal of 400 nm was purified by one 750 short pass filter (Semrock, FF01-750/SP-25), following detected by a monochromator (Acton, Spectra Pro 2300i) attached one CCD (Princeton Instruments, Pixis 100B).

Density functional theory (DFT) calculations

We performed the Berry phase calculations[1, 2] within the DFT framework as implemented in the Vienna ab initio simulation package (VASP).[3, 4] The exchange-correlation interactions were treated within the Perdew-Burke-Ernzerh (PBE) generalized gradient approximation.[5] To complement the deficiencies of DFT in treating dispersion interactions, the third-generation (D3) van der Waals corrections proposed by Grimme[6] was employed. The plane-wave cutoff energy was set to 520 eV, and the k-point mesh to 3 × 3 × 4. The polarization was calculated using a supercell with twice the size of the unit cell so that a centrosymmetric reference phase can be constructed. A convergence threshold of 0.01 eV/Å in force was reached in structural optimization. Electrostatic potential and Mulliken charge were calculated by DMol3[7, 8] code in Materials Studio using a double numerical polarized basis set and PBE[5] exchange-correlation functional.

Density functional theory (DFT) calculations were carried out with the h Berry phase method employed to estimate the microscopic ferroelectric polarization. A dynamic path between the ferroelectric phase and the centrosymmetric reference phase of MNP_3 is established, in which the motion of different components (A, B and X) is desynchronized. Since two MDABCO molecules are required in the simulation cell for this calculation, we constructed a $\sqrt{2} \times \sqrt{2} \times 1$ supercell based on the unit cell of ferroelectric MNP_3. Using this polar structure, the centrosymmetric reference phase is obtained by rotation, displacement and stretch of the components, where two MDABCO molecules in the simulation cell are aligned antiparallel to each other. Starting from the centrosymmetric reference phase (λ = 0), the

coordinate transformation of the constituent atoms is conducted in the following sequence (Fig. 5a): (1) rotation of one MDABCO until both molecules are in the same direction; (2) migration and stretch of PF_6^-; (3) rotation and stretch of NH_4^+; and (4) twist of the rotated MDABCO molecule such that both molecules are in the same conformation. After the above steps, the ferroelectric phase ($\lambda = 1$) is reached. We note that the centre of MDABCO is set at the body centre position of the NH_4^+ sublattice (represented by the N atom).

One important thing to note in Berry phase calculation is that one should construct a path between centrosymmetric to non-centrosymmetric to deal with the branching problem. In principle, Berry phase calculation results only depend on the first and final states. However, corrections have to be made to data points that jump to other branches by the corresponding polarization quantum eR/Ω (e, the charge of the electron; R, lattice vector; Ω, the volume of the simulation cell) to prevent overestimating the polarization values.[9] It is apparent in our raw calculation results (Fig. S13 and S14) that there are many sudden jumps of calculated polarization value as much as polarization quantum, and it should be corrected. Theoretical polarization value of MNI_3 from the original study (27.8 $\mu C/cm^2$)[10] which didn't generate and calculate path, has a gap as much as polarization quantum along [111] direction (19.31 $\mu C/cm^2$) compared with our result (9 $\mu C/cm^2$) and another previously reported result (10.52 $\mu C/cm^2$)[11].

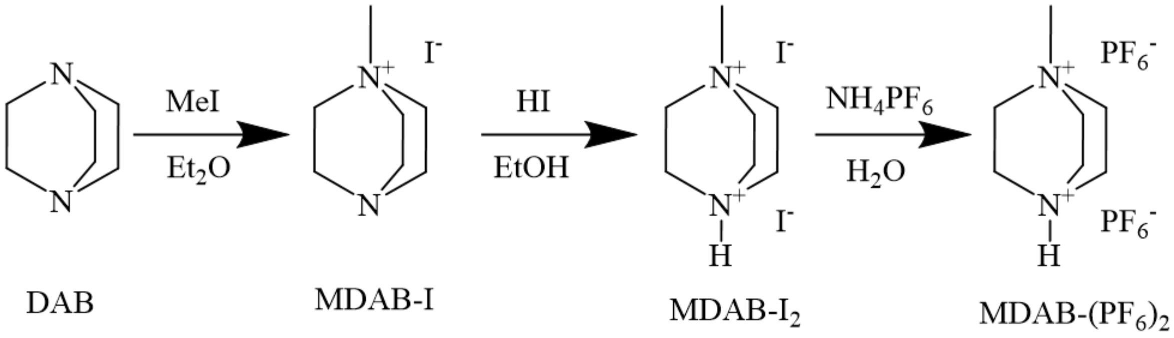

Synthesis of MDABCO-I

13.46 g (0.12 mol) of 1,4-Diazabicyclo[2.2.2]octane (DAB) was dissolved in 500 mL of Et_2O. 50 mL (0.1 mol) of 2 M iodomethane was added dropwise at room temperature while stirring vigorously. The precipitate was filtered to get 18.02 g (0.0709 mol) of MDABCO-I which yield is 70.9%.

^{1}H NMR (DMSO-d_6, 400 MHz): δ (ppm) 2.96 (s, 3H), 3.02 (t, 6H, J = 6.9 Hz), 3.27 (t, 6H, J=8.2 Hz). Elemental analysis found: C 56.75, N 6.11, H 2.76, calculated: C 56.81, N 6.02, H 2.82.

Synthesis of MDABCO-I$_2$

16.24 g (64 mmol) of MDABCO-I was dissolved in ethanol/water 10/1 mixture. 5.29 mL (70 mmol) of HI (1.70 g/ml) was added dropwise at room temperature while stirring vigorously. PrecipitThe precipitatetered and washed with ethanol to get 15.40 g (40 mmol) of MDABCO-I$_2$ which yield is 62.5 %.

^{1}H NMR (DMSO-d_6, 400 MHz): δ (ppm) 3.11 (s, 3H), 3.36 (t, 6H, J = 8.2 Hz), 3.54 (t, 6H, J=7.6 Hz). Elemental analysis found: C 22.33, N 7.29, H 4.22, calculated: C 22.01, N 7.33, H 4.22.

Synthesis of MDABCO-$(PF_6)_2$

3.81 g (10 mmol) of MDABCO-I$_2$ and 9.78 g (60 mmol) of NH_4PF_6 were dissolved in water until saturated each. After mixing of two and vigorously stirred for 3 hours. Precipitated was filtered and the filtrate was treated again with saturated NH_4PF_6 solution until there was no more precipitation. Both preitates were collected and stirred in ethanol for one day to remove NH_4PF_6 and filtered to get 3.08 g (7.37 mmol) of MDABCO-$(PF_6)_2$ which yield was 73.7 %.

^{1}H NMR (DMSO-d_6, 400 MHz): δ (ppm) 3.18 (s, 3H), 3.59 (m, 6H), 3.70 (m, 6H). 13C NMR (DMSO-d$_6$, 400 MHz): δ (ppm) 43.35, 51.49, 52.24. Elemental analysis found: C 20.30, N 6.94,

H 4.33, calculated: C 20.11, N 6.70, H 3.86.

Synthesis of MDABCO-NH_4-$(PF_6)_3$ (MNP_3)

209 mg (0.5 mmol) of MDABCO-$(PF_6)_2$ and 81.5 mg (0.5 mmol) of NH_4PF_6 were dissolved in acetonitrile till saturated, and slow evaporation of solvent form single crystals of MNP_3. Elemental analysis found: C 20.30, N 6.94, H 4.33, calculated: C 20.11, N 6.70, H 3.86.

Table S1. Ionic radii and the Goldschmidt tolerance factors of the corresponding perovskites.

A	B	X	r_A (Å)	r_B (Å)	r_X (Å)	r_A/r_X	r_B/r_X	Goldschmidt tolerance factor
$MDABCO^{2+}$	Rb^+	I^-	2.638	1.49	2.2	1.20	0.677	0.927
$MDABCO^{2+}$	NH_4^+	I^-	2.638	1.46	2.2	1.20	0.664	0.935
$MDABCO^{2+}$	NH_4^+	PF_6^-	2.638	1.46	2.56	1.03	0.570	0.914

Table S2. Crystallographic data for MNP_3 and D-MNP_3

Name	MNP_3	D-MNP_3
Chemical formula	$C_7H_{20}F_{18}N_3P_3$	$C_7H_{19}DF_{18}N_3P_3$
Formula weight	581.17 g/mol	582.16 g/mol
Temperature	RT	RT
Wavelength	0.71073 Å	0.71073 Å
Crystal size	0.356x 0.477 x 0.510 mm	0.235 x 0.268 x 0.436 mm
Crystal system	trigonal	trigonal
Space group	R 3	R 3
a, b, c	a = 10.5847(5) Å<br>b = 10.5847(5) Å<br>c = 14.7547(6) Å	a = 10.614(3) Å<br>b = 10.614(3) Å<br>c = 14.823(3) Å
α , B, γ	α = 90°<br>β = 90°<br>γ = 120°	α = 90°<br>β = 90°<br>γ = 120°
Volume	1431.59(2) $Å^3$	1446.2(8) $Å^3$
Density (calculated)	2.022 g/cm^3	2.002 g/cm^3
Absorption coefficient	0.484 mm^{-1}	0.479 mm^{-1}
F(000)	870	870
R1	0.0318	0.0357
wR2	0.0784	0.0971

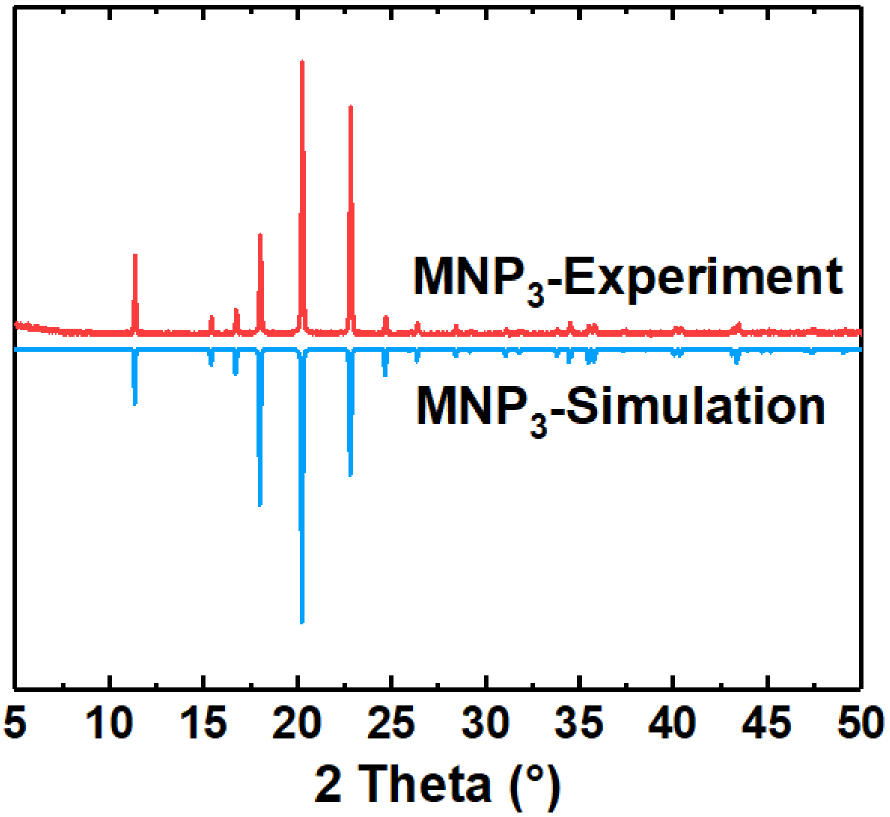

Fig. S1. PXRD result of MNP_3 and comparison with simulation showing high purity of the organic perovskite.

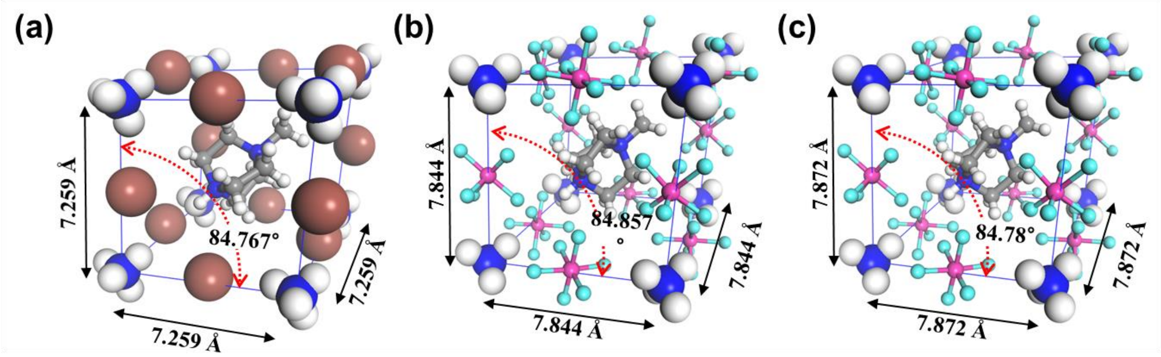

Fig. S2. Unit-cell dimensions of (a) MNI_3, (b) MNP_3, and (c) D-MNP_3.

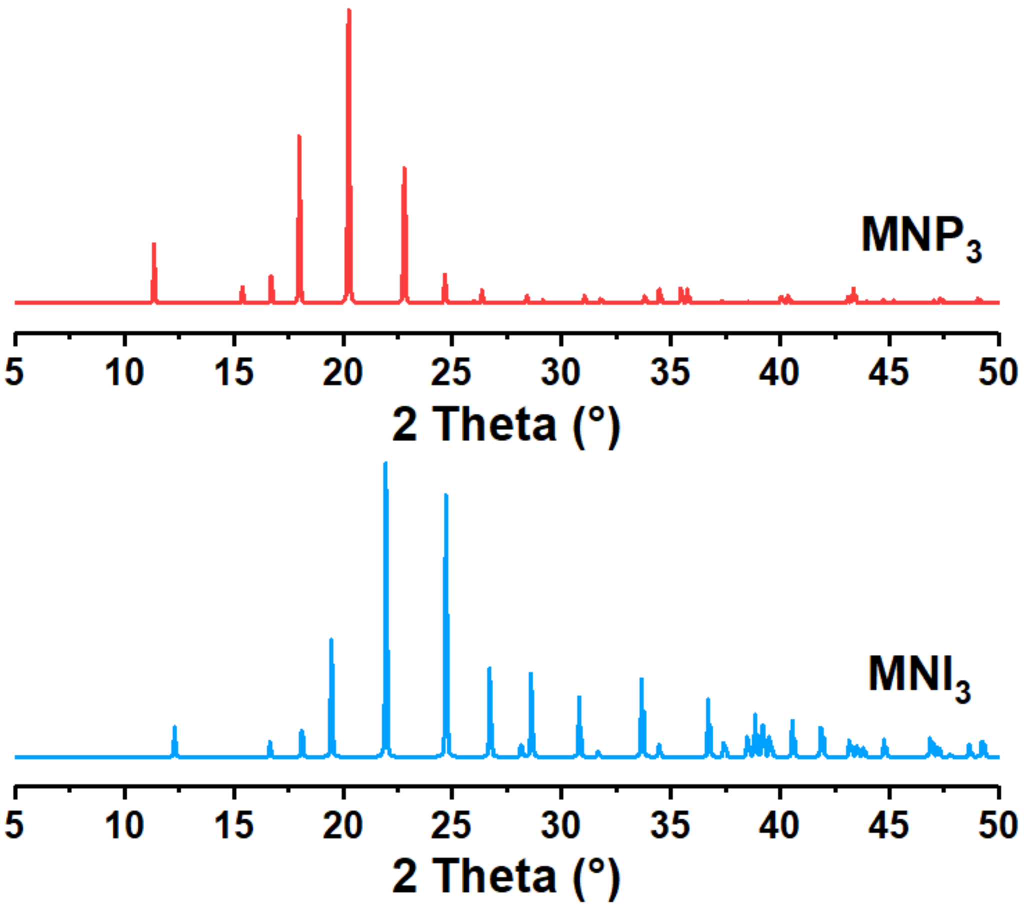

Fig. S3. PXRD results of MNP_3 and MNI_3 show isostructural nature, with MNP_3 having a larger unit cell.

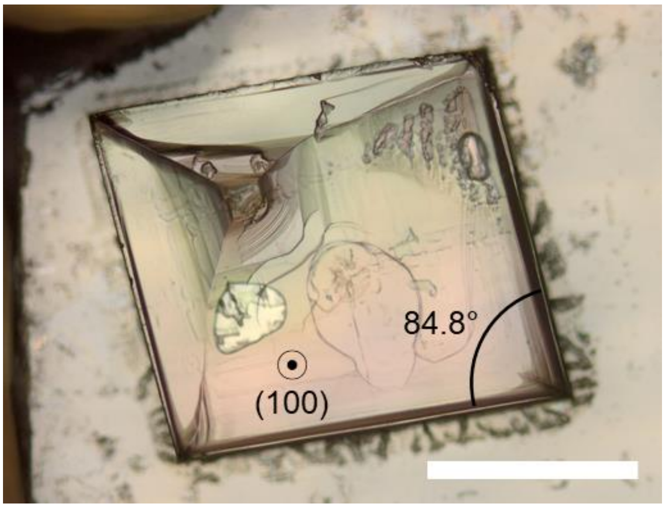

Fig. S4. Optical image of MNP_3 single crystal. Rhombohedral crystal shape follows unit cell of MNP_3 with (100) which is equal to (010), (001) faces. The scale bar is 500 in µm length.

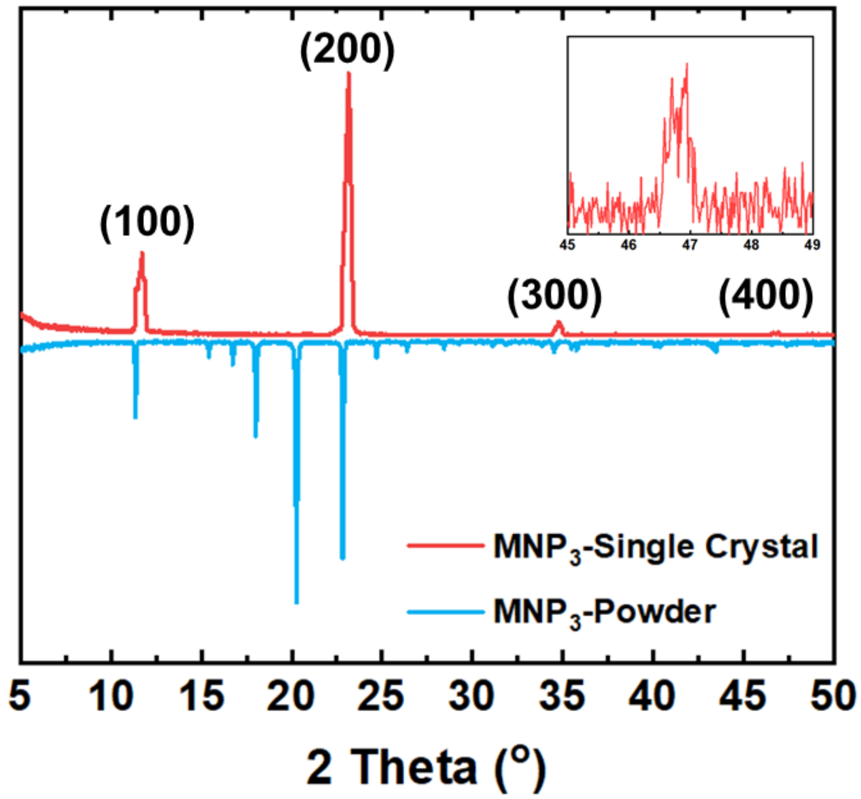

Fig. S5. The out-of-plane XRD pattern for the MNP_3 single crystal showing oriented in (100) plane. Inset is enlarged (400) diffraction peak.

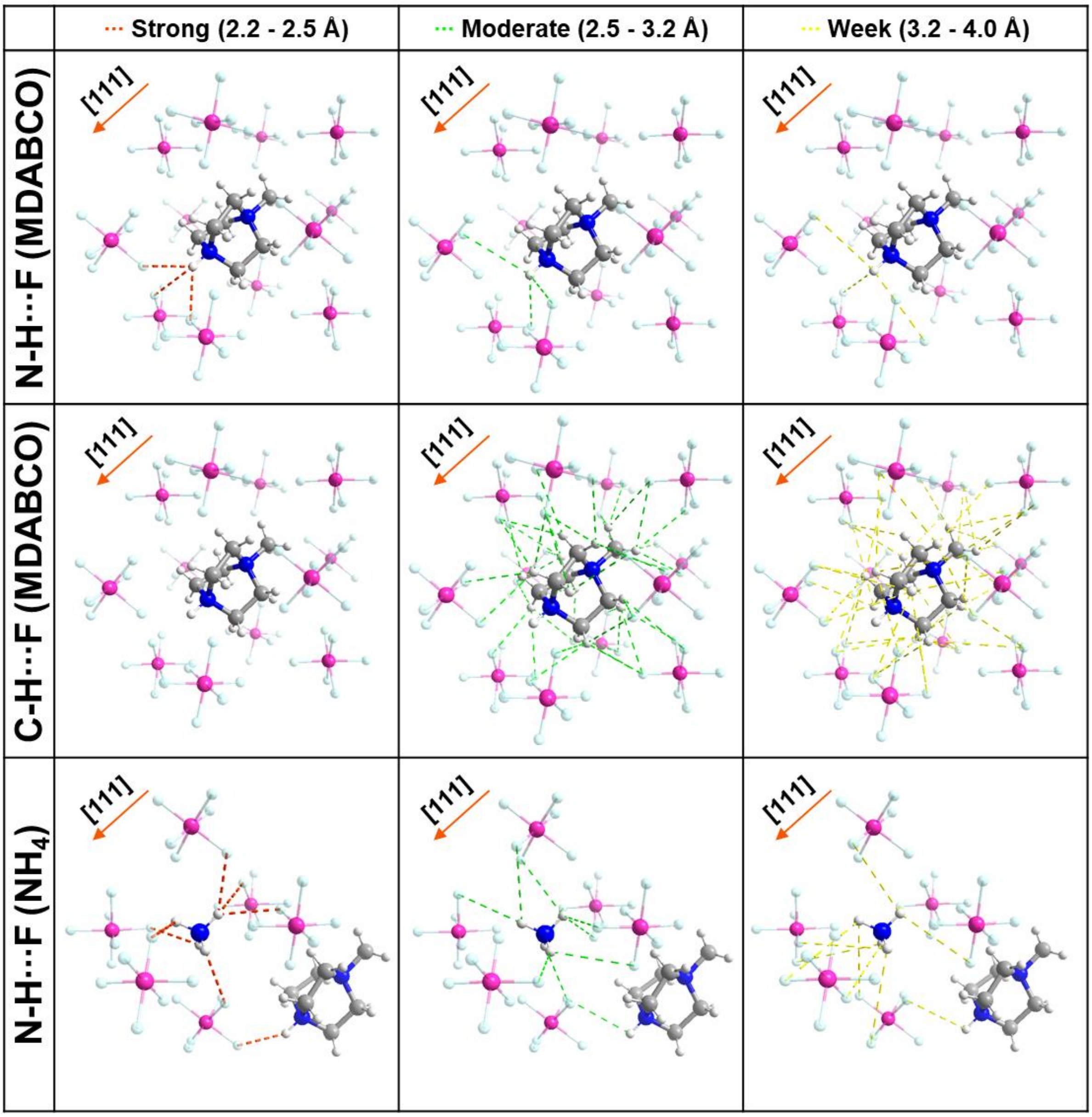

Fig. S6. Hydrogen bonds in MNP_3 crystal sorted by bond length.

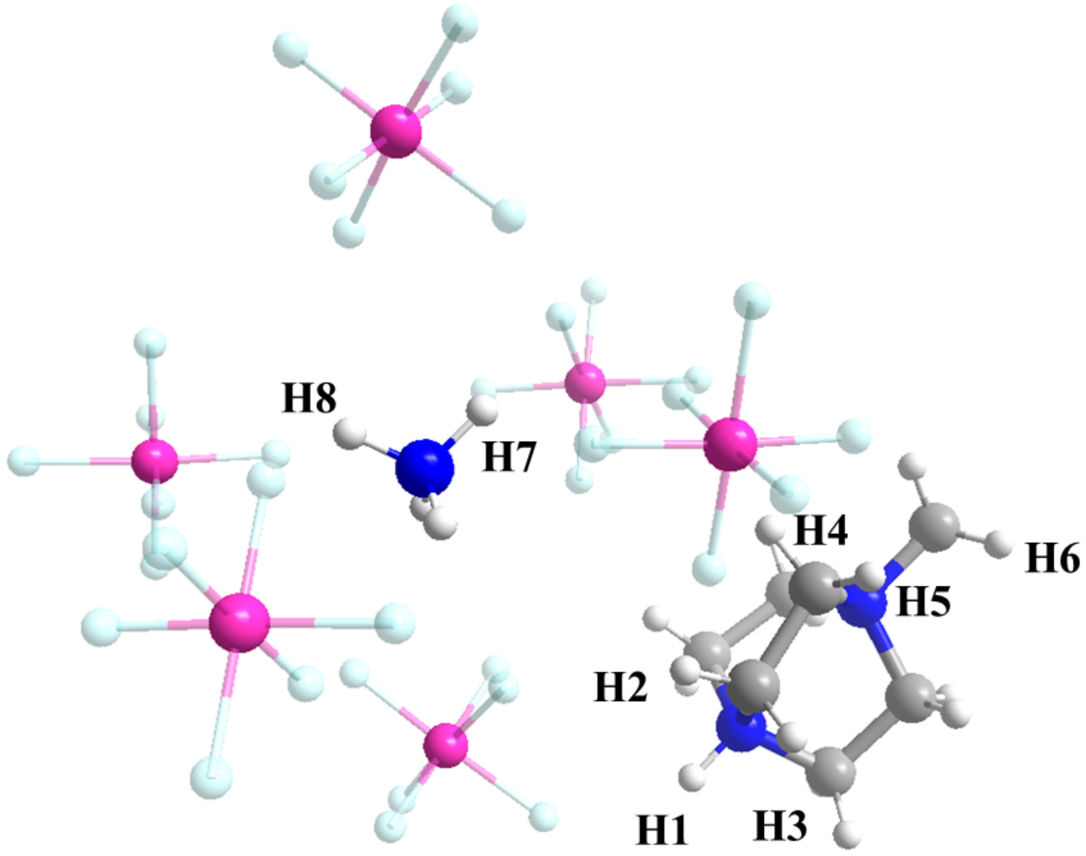

Molecule	H atom	Number of Hydrogen Bond			
		Strong 2.2 - 2.5 Å	Moderate 2.5 - 3.2 Å	Week 3.2 - 4.0 Å	Sum
MDABCO	H1 (NH)	3	3	3	9
	H2 (CH_2)	1×3	4×3	1×3	6×3=18
	H3 (CH_2)	0	3×3	3×3	6×3=18
	H4 (CH_2)	1×3	2×3	2×3	5×3=15
	H5 (CH_2)	0	4×3	2×3	6×3=18
	H6 (CH_3)	0	3×3	2×3	5×3=15
NH_4	H7 (NH)	3	3	3	9
	H8 (NH_3)	1×3	2 ×3	2 ×3	5×3=15
Sum		15	60	42	117

Fig. S7. The number of hydrogen bonds for each hydrogen in MNP_3.

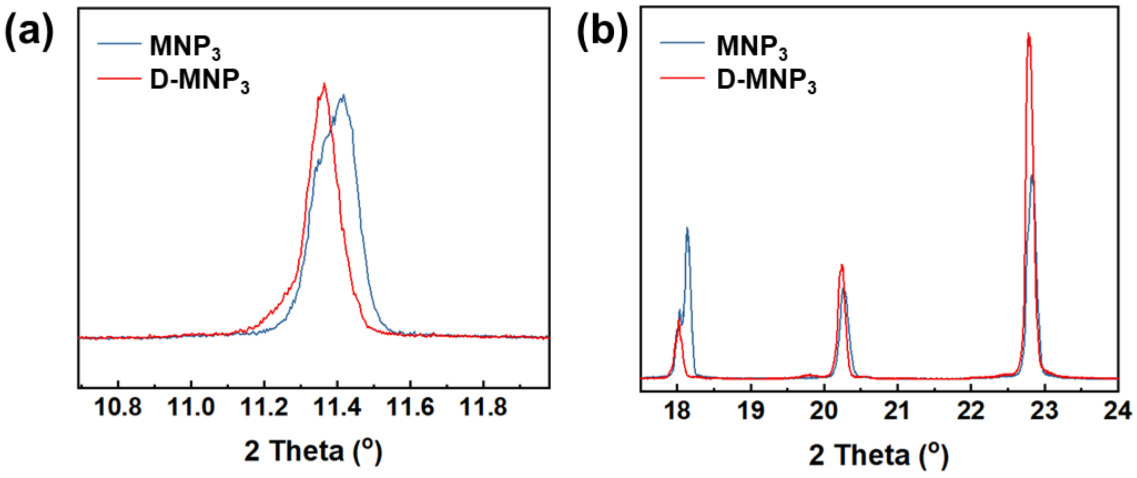

Fig. S8. PXRD comparison between MNP_3 and D-MNP_3. The larger unit cell of D-MNP_3 shows shifted peaks toward a lower angle.

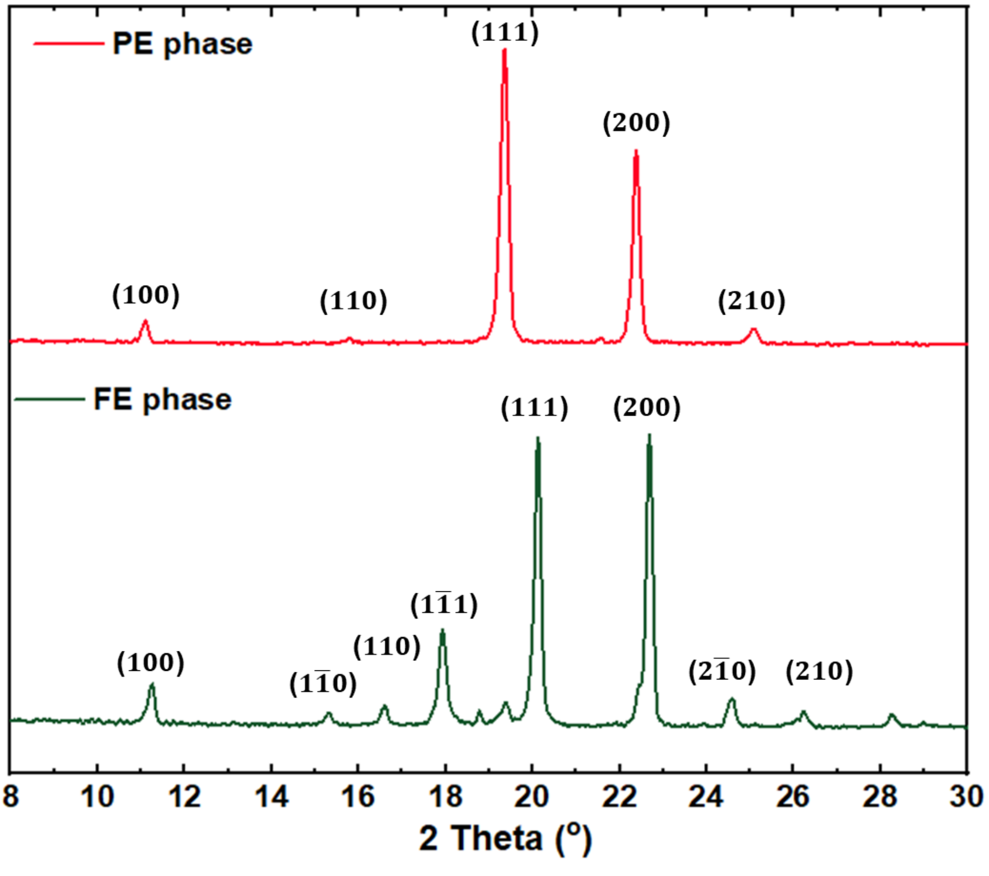

Fig. S9. PXRD comparison between ferroelectric (FE) phase and paraelectric (PE) phase of MNP_3.

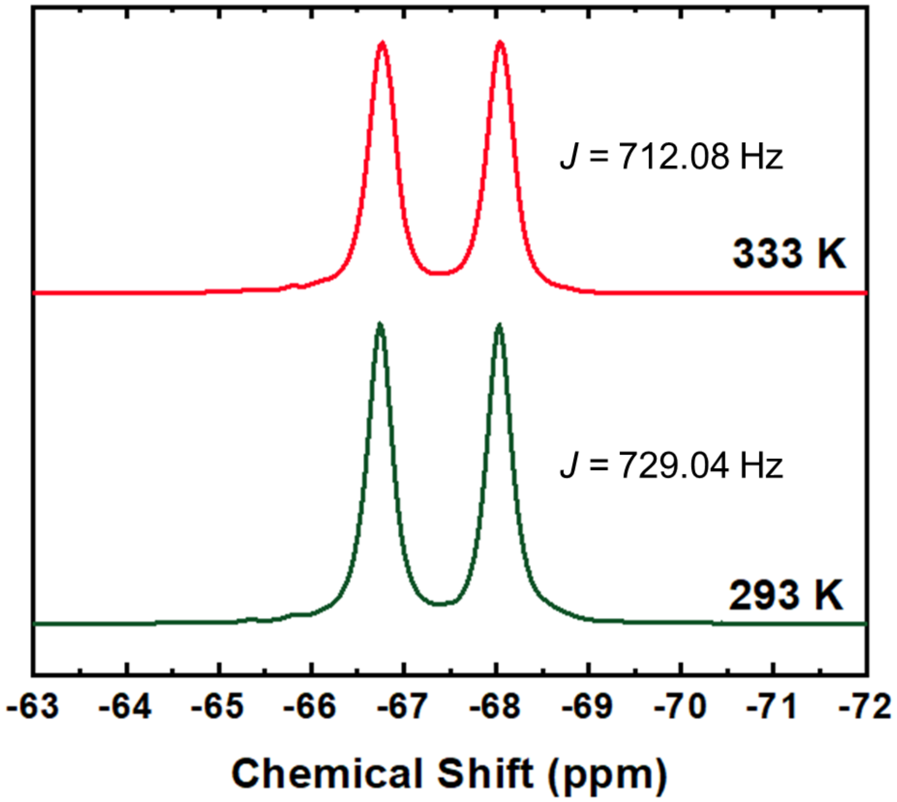

Fig. S10. ^{19}F solid-state NMR spectrum of MNP_3 at 293 K and 333 K. Reduction of J-coupling constant in PE phase (333 K) indicates shrink of P-F distance.

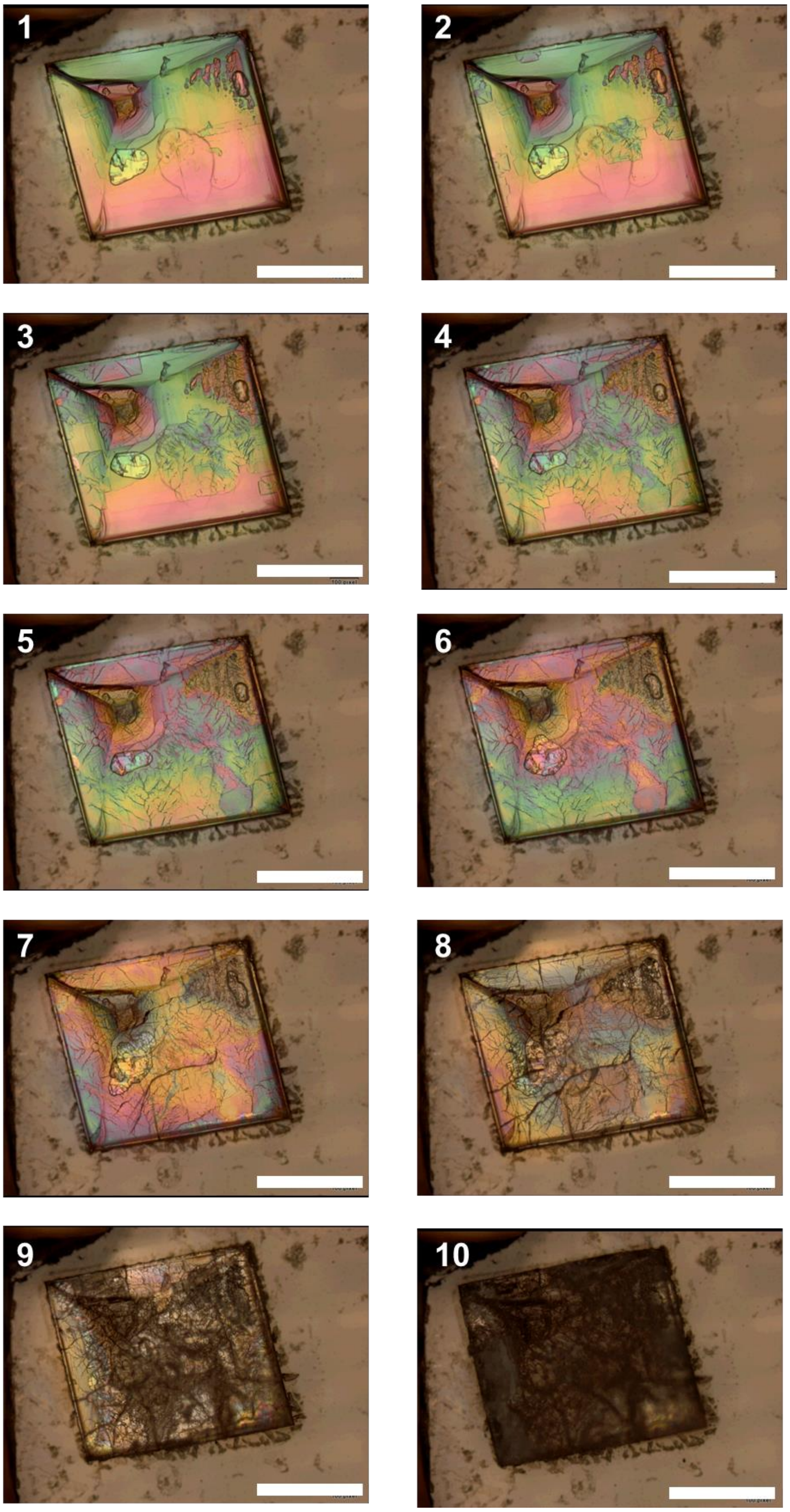

Fig. S11. Polarized light microscopy image of the single crystal of MNP_3 during ferroelectric to paraelectric phase change. The scale bar is 500 μm in length.

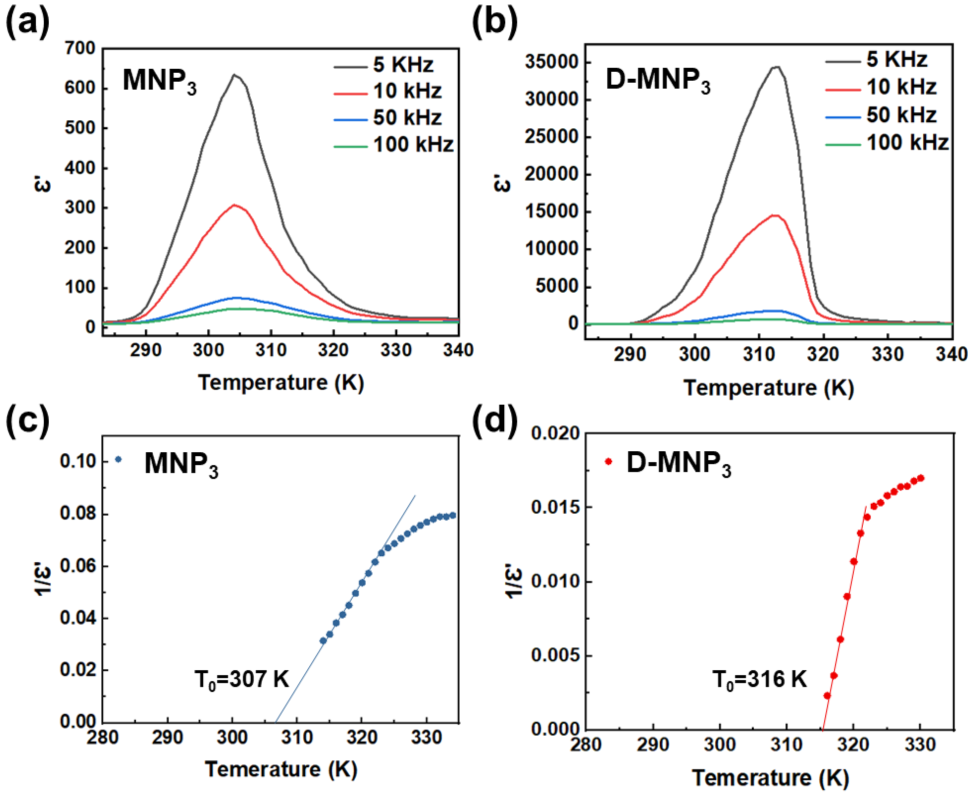

Fig. S12. Temperature-dependent dielectric constants of (a) MNP_3 and (b) D-MNP_3. Fitting of Curie-Weiss law with 100 kHz results of (c) MNP_3 and (d) D-MNP_3.

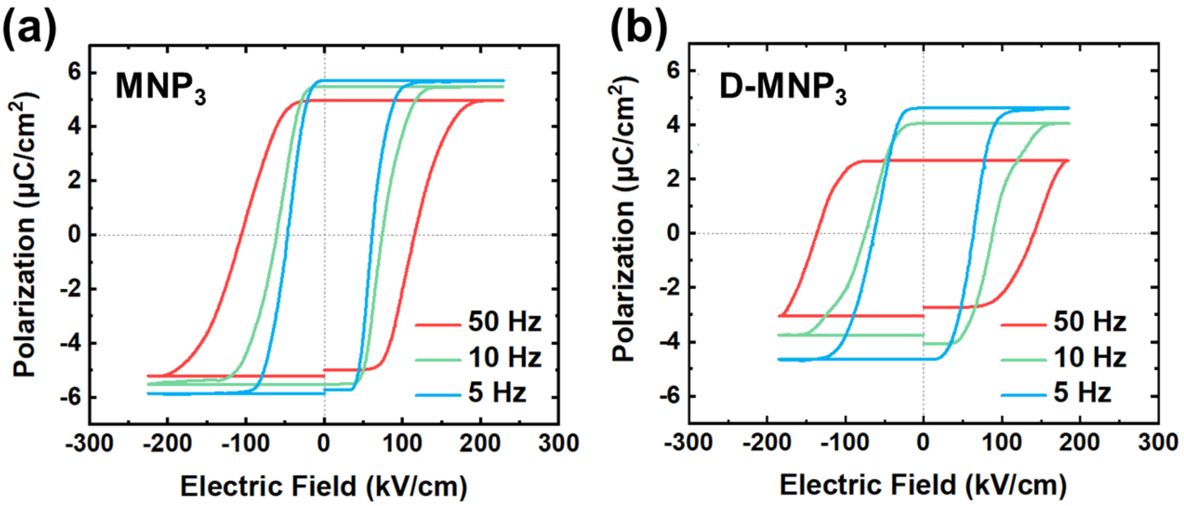

Fig. S13. Polarization (P) and electric field (E) curve (a) MNP_3 and (b) D-MNP_3 with 5, 10, and 50 Hz. D-MNP_3 shows a more reduced polarization value at higher frequencies than MNP_3 due to a stronger hydrogen bond.

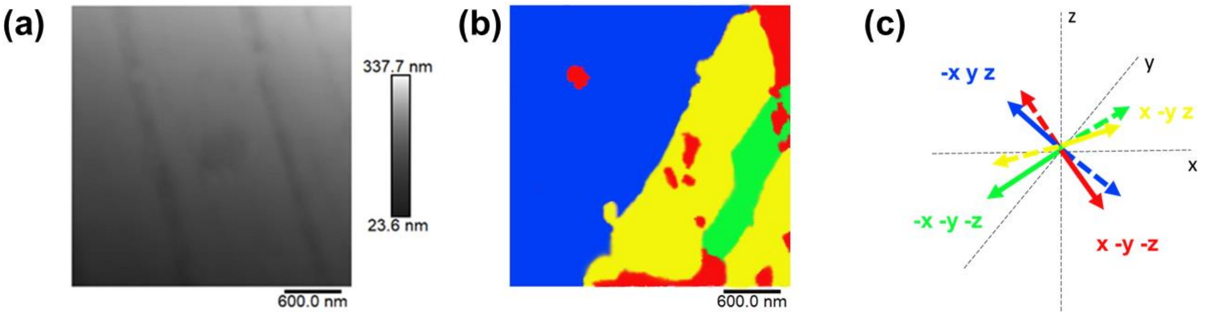

Fig. S14. (a) Topography of MNP_3 by PFM. (b) Areas of different polarization domains. (c) Four different polarization directions according to (b).

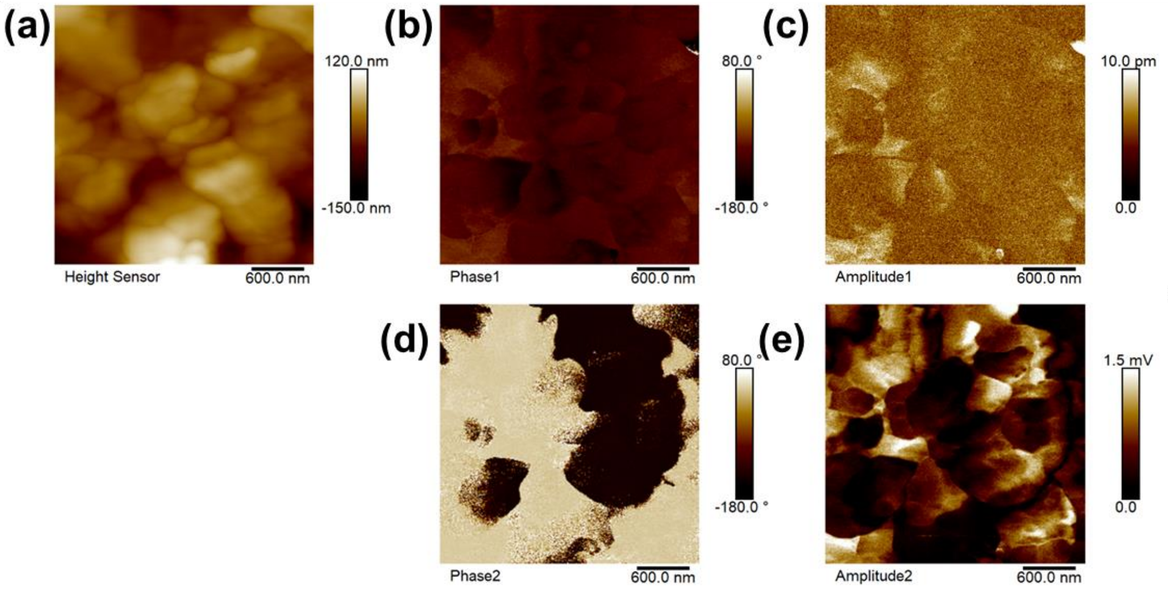

Fig. S15. (a) Topography, (b) out of plain phase, (c) out of plane amplitude, (d) in-plane phase, and (e) in-plain amplitude PFM image of MNP_3 1 μm thick thin film.

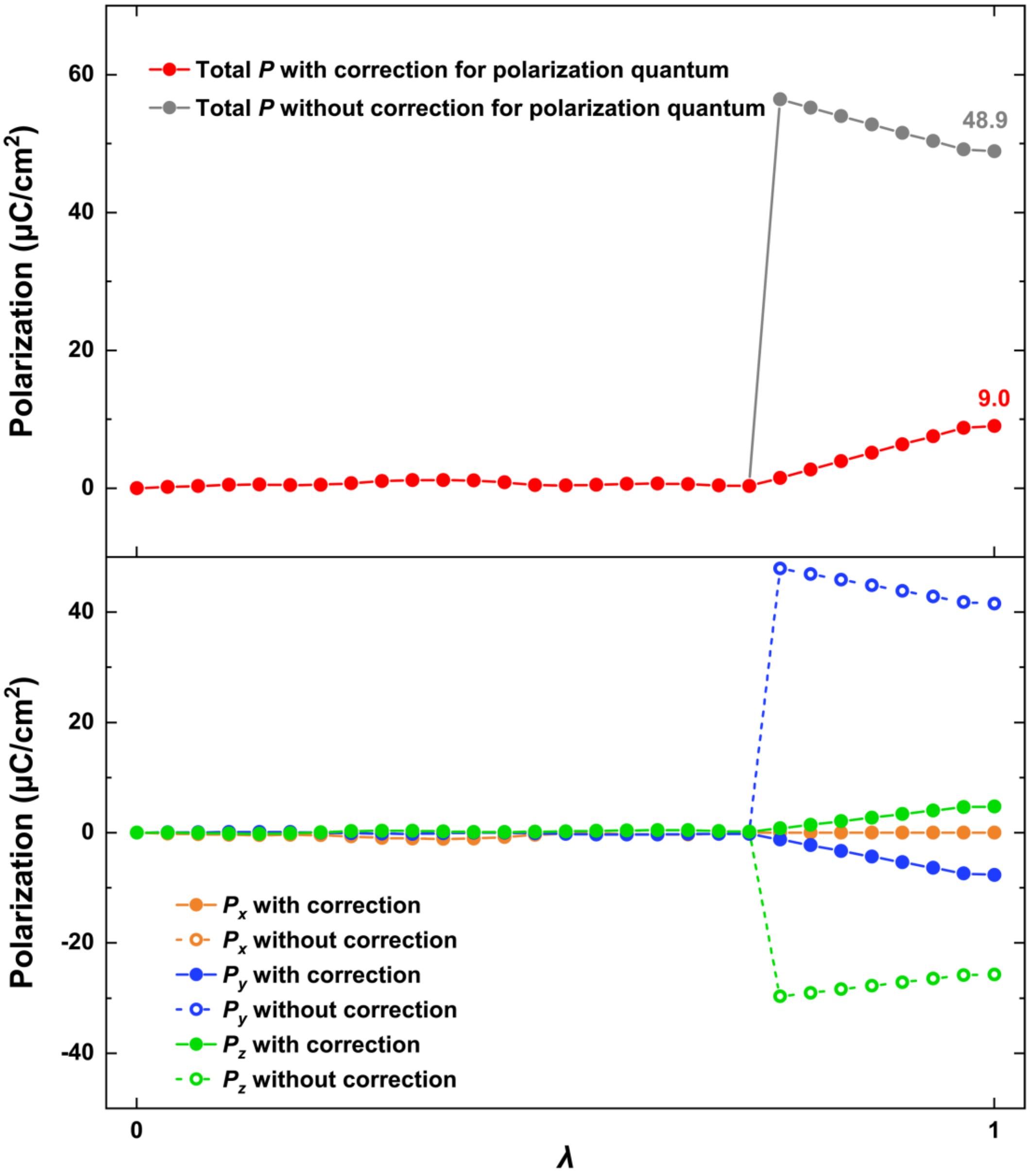

Fig. S16. Berry phase calculation of MNI_3 with and without correction for polarization quantum.

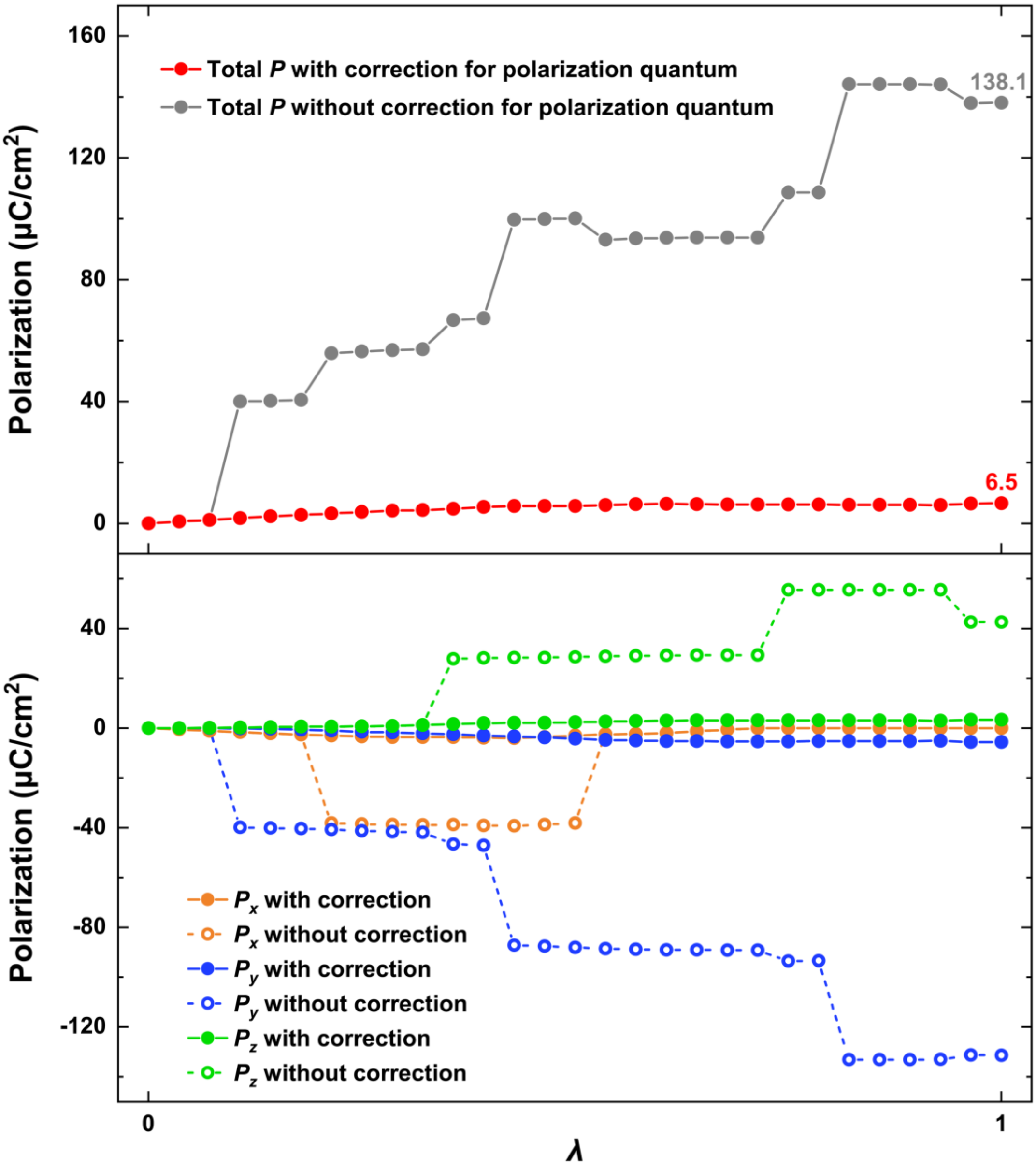

Fig. S17. Berry phase calculation of MNP_3 with and without correction for polarization quantum.

Table S3. Comparison of coercive field and polarization value of representative inorganic, organic, organic-inorganic hybrid and polymer ferroelectrics to MNP_3 and D-MNP_3.

		Coercive Field (kV/cm)	Polarization ($\mu C/cm^2$)
Inorganics	KDP[12]	0.1	4.75
	SnS[13]	20	17.5
	$BaTiO_3$	10	26
	$KNbO_3$	20	30
	HfO_2[14, 15]	1000-2000	10-45
	PbTiO3	7	50
	PZT[16]	21-76	30-55
Organics	DABCO HBF4[17]	15	4.6
	TCAA[18]	4	0.2
	Rochelle salt	0.2	0.25
	Phz-H2ba[19]	0.5	0.8
	Phz-H2ca[19]	0.8	1.8
	Thiourea[20]	0.2	3.2
	[Im]IO_4[21]	20	3.5
	TGS	0.9	3.8
	[H-55dmbp][Hia][22]	2	4.2
	[Im]ClO4[23]	3.5	9.3
	Croconic acid[24]	11	21
	MNI_3[10]	12	22
	[(Me2CH)2NH2]Br[25]	5	23
Hybrid	quinuclidinium [ReO_4][26]	5	3.5
	TMCM-$MnCl_3$[27]	23	4
	[3-Pyrrolinium][$CdCl_3$][28]	2	5.1
	[3-Pyrrolinium][$MnCl_3$][29]	2.5	6.2
	DJP[30]	0.4	9.8
Polymer	P(VDF-TrFE)[31]	500	8
	VDF oligomer[32]	1200	13
	Nylon-5[33]	3000	12.5
	Nylon-11[33]	2000	4.7
This Work	MNP_3	110	5.7
	D-MNP_3	138	4.6

References

1. King-Smith, R. D., Vanderbilt, D. Theory of polarization of crystalline solids. *Physical Review B* **47**, 1651-1654 (1993).
2. Vanderbilt, D., King-Smith, R. D. Electric polarization as a bulk quantity and its relation to surface charge. *Physical Review B* **48**, 4442-4455 (1993).
3. Kresse, G., Furthmüller, J. Efficient iterative schemes for ab initio total-energy calculations using a plane-wave basis set. *Physical Review B* **54**, 11169-11186 (1996).
4. Kresse, G., Furthmüller, J. Efficiency of ab-initio total energy calculations for metals and semiconductors using a plane-wave basis set. *Computational Materials Science* **6**, 15-50 (1996).
5. Perdew, J. P., Burke, K., Ernzerhof, M. Generalized Gradient Approximation Made Simple. *Physical Review Letters* **77**, 3865-3868 (1996).
6. Grimme, S., Antony, J., Ehrlich, S., Krieg, H. A consistent and accurate ab initio parametrization of density functional dispersion correction (DFT-D) for the 94 elements H-Pu. *The Journal of Chemical Physics* **132**, 154104 (2010).
7. Delley, B. An all-electron numerical method for solving the local density functional for polyatomic molecules. *The Journal of Chemical Physics* **92**, 508-517 (1990).
8. Delley, B. From molecules to solids with the DMol3 approach. *The Journal of Chemical Physics* **113**, 7756-7764 (2000).
9. Spaldin, N. A. A beginner's guide to the modern theory of polarization. *Journal of Solid State Chemistry* **195**, 2-10 (2012).
10. Ye, H.-Y*., et al.* Metal-free three-dimensional perovskite ferroelectrics. *Science* **361**, 151 (2018).
11. Wang, H*., et al.* Large piezoelectric response in a family of metal-free perovskite ferroelectric compounds from first-principles calculations. *npj Computational Materials* **5**, 17 (2019).
12. Koval, S., Kohanoff, J., Migoni, R. L., Tosatti, E. Ferroelectricity and Isotope Effects in Hydrogen-Bonded KDP Crystals. *Physical Review Letters* **89**, 187602 (2002).
13. Kwon, K. C*., et al.* In-Plane Ferroelectric Tin Monosulfide and Its Application in a Ferroelectric Analog Synaptic Device. *ACS Nano* **14**, 7628-7638 (2020).
14. Muller, J., Polakowski, P., Riedel, S., Mueller, S., Yurchuk, E., Mikolajick, T. Ferroelectric Hafnium Oxide A Game Changer to FRAM? *2014 14th Annual Non-Volatile Memory Technology Symposium (NVMTS)*, 1-7 (2014).
15. Polakowski, P., Müller, J. Ferroelectricity in undoped hafnium oxide. *Applied Physics Letters* **106**, 232905 (2015).
16. Foster, C. M*., et al.* Single-crystal Pb(ZrxTi1−x)O3 thin films prepared by metal-organic chemical vapor deposition: Systematic compositional variation of electronic and optical properties. *Journal of Applied Physics* **81**, 2349-2357 (1997).
17. Shi, P.-P., Tang, Y.-Y., Li, P.-F., Ye, H.-Y., Xiong, R.-G. De Novo Discovery of [Hdabco]BF4 Molecular Ferroelectric Thin Film for Nonvolatile Low-Voltage Memories. *Journal of the American Chemical Society* **139**, 1319-1324 (2017).
18. Kamishina, Y., Akishige, Y., Hashimoto, M. Ferroelectric Activity on Organic Crystal

Trichloroacetamide. *Journal of the Physical Society of Japan* **60**, 2147-2150 (1991).

19. Horiuchi, S., Tokura, Y. Organic ferroelectrics. *Nature Materials* **7**, 357-366 (2008).
20. Goldsmith, G. J., White, J. G. Ferroelectric Behavior of Thiourea. *The Journal of Chemical Physics* **31**, 1175-1187 (1959).
21. Zhang, Y.*, et al.* Switchable Dielectric, Piezoelectric, and Second-Harmonic Generation Bistability in a New Improper Ferroelectric above Room Temperature. *Advanced Materials* **26**, 4515-4520 (2014).
22. Horiuchi, S., Kumai, R., Tokura, Y. A Supramolecular Ferroelectric Realized by Collective Proton Transfer. *Angewandte Chemie International Edition* **46**, 3497-3501 (2007).
23. Zhang, Y.*, et al.* A Molecular Ferroelectric Thin Film of Imidazolium Perchlorate That Shows Superior Electromechanical Coupling. *Angewandte Chemie International Edition* **53**, 5064-5068 (2014).
24. Horiuchi, S.*, et al.* Above-room-temperature ferroelectricity in a single-component molecular crystal. *Nature* **463**, 789-792 (2010).
25. Fu, D.-W.*, et al.* Diisopropylammonium Bromide Is a High-Temperature Molecular Ferroelectric Crystal. *Science* **339**, 425 (2013).
26. Harada, J.*, et al.* Directionally tunable and mechanically deformable ferroelectric crystals from rotating polar globular ionic molecules. *Nature Chemistry* **8**, 946-952 (2016).
27. You, Y.-M.*, et al.* An organic-inorganic perovskite ferroelectric with large piezoelectric response. *Science* **357**, 306 (2017).
28. Ye, H.-Y., Zhang, Y., Fu, D.-W., Xiong, R.-G. An Above-Room-Temperature Ferroelectric Organo–Metal Halide Perovskite: (3-Pyrrolinium)(CdCl3). *Angewandte Chemie International Edition* **53**, 11242-11247 (2014).
29. Ye, H.-Y.*, et al.* High-Temperature Ferroelectricity and Photoluminescence in a Hybrid Organic–Inorganic Compound: (3-Pyrrolinium)MnCl3. *Journal of the American Chemical Society* **137**, 13148-13154 (2015).
30. Park, I.-H.*, et al.* Ferroelectricity and Rashba Effect in a Two-Dimensional Dion-Jacobson Hybrid Organic–Inorganic Perovskite. *Journal of the American Chemical Society* **141**, 15972-15976 (2019).
31. Chen, X., Han, X., Shen, Q.-D. PVDF-Based Ferroelectric Polymers in Modern Flexible Electronics. *Advanced Electronic Materials* **3**, 1600460 (2017).
32. Noda, K., Ishida, K., Kubono, A., Horiuchi, T., Yamada, H., Matsushige, K. Remanent polarization of evaporated films of vinylidene fluoride oligomers. *Journal of Applied Physics* **93**, 2866-2870 (2003).
33. Anwar, S.*, et al.* Solution-processed transparent ferroelectric nylon thin films. *Science Advances* **5**, eaav3489 (2019).
